# Deciphering cellular signals in adult mouse sinoatrial node cells

**DOI:** 10.1016/j.isci.2021.103693

**Published:** 2021-12-25

**Authors:** Gopireddy R. Reddy, Lu Ren, Phung N. Thai, Jessica L. Caldwell, Manuela Zaccolo, Julie Bossuyt, Crystal M. Ripplinger, Yang K. Xiang, Madeline Nieves-Cintrón, Nipavan Chiamvimonvat, Manuel F. Navedo

**Affiliations:** 1 Department of Pharmacology, University of California Davis, One Shields Avenue MED: PHARM Tupper 242, Davis, CA 95616, USA; 2 Department of Internal Medicine, University of California Davis, 451 Health Science Drive, GBSF 6315, Davis, CA 95616, USA; 3 Department of Physiology, Anatomy and Genetics, University of Oxford, Oxford, OX1 3PT, UK; 4 VA Northern California Healthcare System, 10535 Hospital Way, Mather, CA 95655, USA

**Keywords:** Cell biology, Functional aspects of cell biology, Biology experimental methods

## Abstract

Sinoatrial node (SAN) cells are the pacemakers of the heart. This study describes a method for culturing and infection of adult mouse SAN cells with FRET-based biosensors that can be exploited to examine signaling events. SAN cells cultured in media with blebbistatin or (S)-nitro-blebbistatin retain their morphology, protein distribution, action potential (AP) waveform, and cAMP dynamics for at least 40 h. SAN cells expressing targeted cAMP sensors show distinct β-adrenergic-mediated cAMP pools. Cyclic GMP, protein kinase A, Ca^2+^/CaM kinase II, and protein kinase D in SAN cells also show unique dynamics to different stimuli. Heart failure SAN cells show a decrease in cAMP and cGMP levels. In summary, a reliable method for maintaining adult mouse SAN cells in culture is presented, which facilitates studies of signaling networks and regulatory mechanisms during physiological and pathological conditions.

## Highlights

• Optimized culturing method of adult SAN cells from healthy and disease-state mice
• Cultured adult SAN cells can be infected with diverse fluorescence biosensors
• Examination of signaling networks, including discrete cAMP pools, in adult SAN cells
• SAN cells cAMP and cGMP signals are impaired during heart failure

## Graphical abstract

**Figure:**
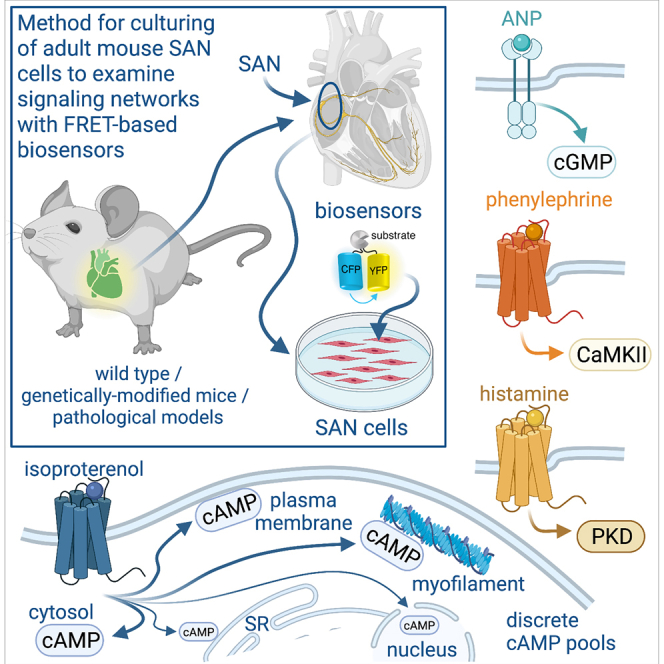

## Introduction

Sinoatrial node (SAN) cells are the intrinsic pacemakers responsible for normal heart rhythm. Current evidence suggests that SAN cell automaticity is controlled by an interdependent membrane clock and a Ca^2+^ clock (Lakatta et al., 2010; Yaniv et al., 2015b). The membrane clock is driven by ion channels and transporters at the plasma membrane (PM), whereas spontaneous Ca^2+^ release events originating from the sarcoplasmic reticulum (SR) drive the Ca^2+^ clock (Lakatta et al., 2010; Yaniv et al., 2015b). The cross talk and synergy between these clocks are modulated by second messengers and signaling molecules acting on coupled-clock proteins (Behar and Yaniv, 2016; Han et al., 1995; Liao et al., 2010; Shimizu et al., 2002; van Borren et al., 2010; Vinogradova et al., 2000; Yaniv et al., 2013, 2015a, 2015b). This regulation may be altered during pathological conditions (Neco et al., 2012; Verkerk et al., 2003). Among the second messengers, cyclic adenosine monophosphate (cAMP) and the nitric oxide (NO)/cyclic guanosine monophosphate (cGMP) axis have been shown to modulate coupled-clock proteins that control SAN pacemaking activity (DiFrancesco and Borer, 2007; Han et al., 1995; Musialek et al., 1997; Peters et al., 2020; Shimizu et al., 2002; Vinogradova et al., 2018b; Yaniv et al., 2015b). Coupled-clock proteins can also be regulated by protein kinase A (PKA) (Behar and Yaniv, 2016; Liao et al., 2010; Vinogradova and Lakatta, 2009; Yaniv et al., 2015a), Ca^2+^/calmodulin-dependent protein kinase II (CaMKII) (Vinogradova et al., 2000; Wu and Anderson, 2014; Wu et al., 2009; Xie et al., 2015; Yaniv et al., 2013), and protein kinase D (PKD) (Wood and Bossuyt, 2017), although SAN regulation by PKD is unclear. In all cases, the spatiotemporal dynamics of these second messengers and signaling molecules in SAN cells are poorly understood. Addressing this issue is important to gain insight into how complex signaling networks regulate SAN pacemaking activity in health and disease (MacDonald et al., 2020).

Understanding the spatiotemporal dynamics of second messengers and signaling molecules in live cells has been made possible, at least in part, by the development of genetically encoded Förster resonance energy transfer (FRET) based biosensors (Greenwald et al., 2018). A number of these biosensors have been created to measure cAMP and cGMP signaling as well as PKA, CaMKII, and PKD activity in live cells. The implementation of this technology depends on infecting cells with the biosensor of interest, as there is limited availability of transgenic animals expressing these reporters. However, the use of biosensors in SAN cells may be challenging given that isolated cells need to be infected and cultured for at least 40 h to achieve optimal levels of biosensor expression for accurate quantification of the FRET signal (Reddy et al., 2018). Moreover, under certain culturing conditions, SAN cells undergo a rapid remodeling that alters their electrical signals and morphology (Segal et al., 2019; Yang et al., 2012), which may impact the accurate assessment of the spatiotemporal dynamics of signaling networks. Thus, a method is necessary to culture SAN cells under conditions in which their functional properties and morphology are minimally impacted (Kirschner Peretz et al., 2020) while allowing for the expression of genetically encoded biosensors.

Prior studies suggest that contraction uncouplers, such as 2,3-butanedione 2-monoxime (BDM) or blebbistatin, may help preserve morphology and functional properties of cultured cardiac cells (Kabaeva et al., 2008; Kirschner Peretz et al., 2017, 2020; Kivisto et al., 1995; Liu et al., 1996; Muramatsu et al., 1996; Reddy et al., 2018; Segal et al., 2019; St Clair et al., 2015; Yang et al., 2012). However, the efficiency of these treatments, especially BDM and blebbistatin, for maintaining a large population of adult mouse SAN cells in culture is unclear, as prior reports have shown examples of only one isolated cell per culturing condition (Kirschner Peretz et al., 2020; Segal et al., 2019; St Clair et al., 2015). In one study, it was reported that at least 13% of the rabbit SAN cells using a culturing method with 25 μM blebbistatin lost their spindle-shaped morphology and developed some projections (Segal et al., 2019). Moreover, although reports have shown that infection of cultured mouse and rabbit SAN cells with a fluorescence probe (e.g., GFP, mCherry) is possible (Segal et al., 2019; St Clair et al., 2015), the functional significance was never tested.

Here, we describe a method for maintaining adult mouse SAN cells in culture. This method is based on supplementation of the culture media with blebbistatin/(S)-nitro blebbistatin. The method was found to preserve cell length and morphology in a larger population of cultured SAN cells than previously reported. In addition, protein distribution, action potential (AP), and cAMP signaling properties were conserved in cultured SAN cells for at least 40 h. The method can be easily implemented for the study of SAN cells from genetically modified mice or pathological mouse models. It also facilitates the robust functional expression of exogenous probes and proteins, including FRET-based biosensors to study signaling networks in SAN cells from adult mouse models, and likely other species, during physiological and pathological conditions. By exploiting this method, the current study provides insight into cAMP and cGMP signaling as well as PKA, CaMKII, and PKD activity in mouse SAN cells. The study further describes how second messengers (cAMP and cGMP) are altered in mouse SAN cells during heart failure (HF). Thus, this method opens opportunities for further understanding how signaling networks integrate into and regulate critical components of the coupled-clock system of SAN cells in health and disease.

## Results

**Figure 1:**
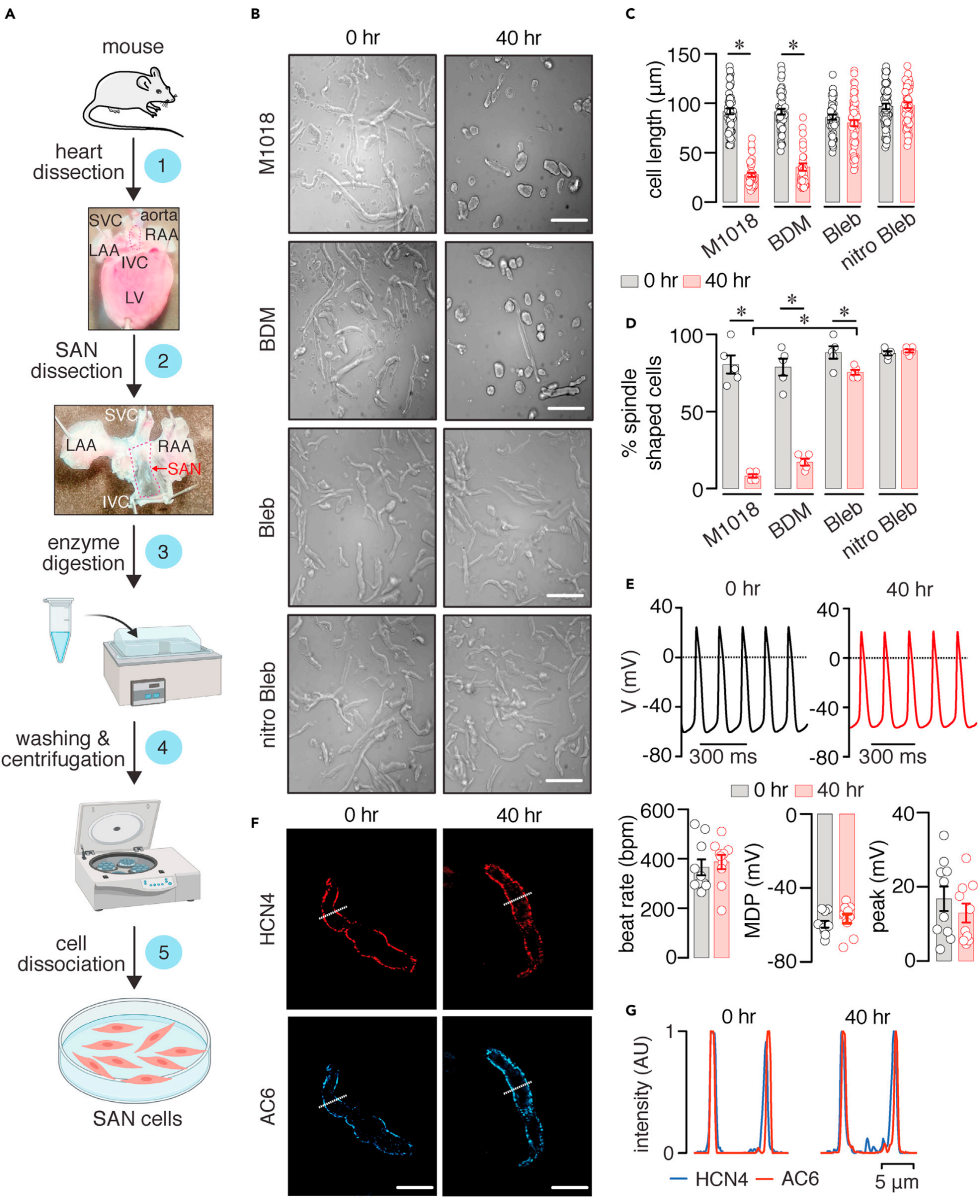
Design and validation of the adult mouse SAN cell culturing method (A) Steps for obtaining and culturing adult mouse SAN cells. Some of the images were created with Biorender.com. (B–D) Brightfield images of adult mouse SAN cells in M1018 media alone or containing 10 mM 2,3-Butanedione 2-monoxime (BDM), 6.5 μM blebbistatin (Bleb), or 20 μM (S)-nitro blebbistatin (nitro Bleb) at 0 and 40 h (hr) in culture. Scale bar, 10 mm. Scatterplots of cell length (C; n = >48 cells from three preparations per condition) and % spindle-shaped cells (D; n = ≥5 images from three preparations per condition) in the different media conditions at 0 and 40 h in culture. Statistical differences were assessed with two-tailed Kruskal-Wallis with Dunn's test for comparison between all groups and two-tailed Mann-Whitney test for comparisons between the 0 and 40 h groups. Exact p values are available in Table S1. (E) Representative spontaneous action potential (AP) traces and scatterplot of firing rate, maximum diastolic potential (MDP), and peak amplitude obtained from adult mouse SAN cells freshly dissociated or cultured in M1018 media +20 μM (S)-nitro blebbistatin for 40 h (n = 10 cells per condition). AP recordings were performed after removing the M1018 + (S)-nitro blebbistatin media and washing cells with control recording solution. P = 0.5657 for firing rate comparison, 0.2393 for MDP comparison, and 0.6905 for peak amplitude comparison between 0 and 40 h datasets with two-tailed Mann-Whitney test. (F) Representative images of the fluorescence associated with HCN4 or AC6 in adult mouse SAN cells at 0 and 40 h in culture (similar results were observed in >5 cells from two preparations). Scale bar, 10 μm. (G) Line intensity profiles obtained from the regions highlighted by the dotted lines in the representative images. Significance (∗) was considered at P < 0.05. Data represent mean ± SEM.

Figure 1A illustrates the steps for obtaining adult mouse SAN cells for culture. The mouse hearts are excised as described in the STAR Methods section (step 1) (Fenske et al., 2016; Mangoni and Nargeot, 2001; Sharpe et al., 2016; Vinogradova et al., 2000; Zhang et al., 2002). SAN tissue is collected from a region within previously identified landmark structures, including the superior and inferior vena cava, crista terminalis, and atrial septum (St Clair et al., 2015; Zhang et al., 2002) (step 2). The SAN tissue is enzymatically digested (step 3) and washed via centrifugation (step 4). Finally, SAN cells are dispersed and plated on coverslips (step 5) for long-term culturing and infection.

### Blebbistatin preserves adult mouse SAN cell morphology, AP, and protein distribution

Adult mouse SAN cells were cultured in M1018 media (control) or M1018 media supplemented with 10 mM BDM, 6.5 μM blebbistatin, or 20 μM (S)-nitro blebbistatin (Figure 1B). The BDM concentration was selected based on a prior study using this compound in SAN cells (St Clair et al., 2015). Blebbistatin and (S)-nitro blebbistatin were used at concentrations recently found to be optimal for culturing ventricular myocytes (Reddy et al., 2018). Images were collected from a broad field of view containing multiple cells. SAN cells showed a similar length range (∼86–97 μm) and cell shape (e.g., spindle-shaped versus spherical) at 0 h in culture for all conditions (Figures 1C and 1D and Table S1). After 40 h in culture, SAN cells in media with M1018 and BDM exhibited a significant reduction in cell length and percentage of spindle-shaped cells compared with the same condition at 0 h (Figures 1C and 1D and Table S1). In contrast, SAN cells cultured in media containing blebbistatin or (S)-nitro blebbistatin for 40 h maintained their cell length compared with 0-h cultured cells (Figure 1C and Table S1). The percentage of spindle-shaped cells was slightly reduced in cells cultured with blebbistatin compared with the 0 h condition (Figure 1D and Table S1). Blebbistatin was still more effective at maintaining cell shape than just culturing cells in M1018 media alone (Table S1). Cell shape was similar in SAN cells cultured in (S)-nitro blebbistatin for 0 and 40 h (Figure 1D and Table S1). These results suggest that blebbistatin and (S)-nitro blebbistatin help retain SAN cell length and shape in a larger number of cultured cells for at least 40 h, compared with M1018 and BDM. Moreover, the blebbistatin/(S)-nitro blebbistatin concentrations seem to be effective for culturing of adult mouse SAN cells.

Blebbistatin has been shown to increase autofluorescence in cultured ventricular myocytes, and this is significantly reduced with the use of (S)-nitro blebbistatin (Reddy et al., 2018). This is important for the accurate acquisition of fluorescent signals, such as those from FRET biosensors. Because of this and the slightly better cell morphology preservation, the rest of the experiments were performed using adult mouse SAN cells cultured in media supplemented with (S)-nitro blebbistatin.

To determine if 20 μM (S)-nitro blebbistatin helps preserve adult mouse SAN pacemaking activity, we recorded APs in SAN cells at 0 and 40 h in culture. (S)-nitro blebbistatin was washed out before AP recordings. Results show that the AP beat rate, mean diastolic potential (MDP), and peak amplitude are similar in SAN cells at 0 and 40 h in culture with (S)-nitro blebbistatin (Figure 1E). In subsequent experiments, confocal immunofluorescent imaging of the hyperpolarization-activated cyclic nucleotide-gated 4 (HCN4) channel and adenylyl cyclase 6 (AC6) revealed similar distribution between these proteins at the membrane in SAN cells at 0 and 40 h in culture with (S)-nitro blebbistatin (Figures 1F and 1G). These results suggest that (S)-nitro blebbistatin helps maintain the AP waveform and protein distribution in culture for at least 40 h.

### Expression of FRET biosensors in adult mouse SAN cells

**Figure 2:**
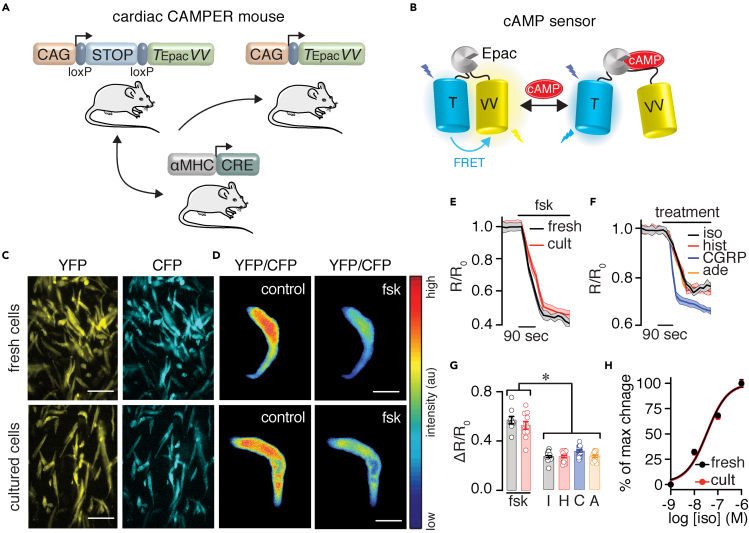
cAMP signals in freshly isolated and cultured CAMPER_CM_ SAN cells (A) Cartoon showing the strategy for the generation of the CAMPER_CM_ mouse. (B) Diagram of the cAMP biosensor expressed in the CAMPER_CM_ mouse in the cAMP-free and cAMP-bound form. (C) Representative population of freshly isolated and cultured adult SAN cells from the CAMPER_CM_ mouse displaying YFP and CFP fluorescence (similar results were observed in two independent preparations). Scale bar, 10 mm. (D) Exemplary pseudo-colored FRET ratio images of freshly isolated and cultured CAMPER_CM_ SAN cells before and after 10 μM forskolin (fsk). Scale bar, 10 μm. (E) Average FRET ratio traces (mean, solid lines; SEM, shadow) before and after application of 10 μM fsk in freshly isolated (fresh) and cultured (cult) CAMPER_CM_ SAN cells. (F) Average FRET ratio traces (mean, solid lines; SEM, shadow) before and after application of 100 nM isoproterenol (iso), 1 μM histamine (hist), 1 nM CGRP, and 1 μM adenosine (ade). (G) Scatterplot summarizing freshly dissociated and cultured CAMPER_CM_ SAN cells ΔR/R_0_ FRET responses to fsk (n = >9 cells from two preparations per condition; P = 0.295 with two-tailed Mann-Whitney test for comparison of fsk responses in freshly dissociated and cultured CAMPER_CM_ SAN cells), as well as ΔR/R_0_ FRET responses to iso, I; hist, H; CGRP, C; and ade, A in cultured CAMPER_CM_ cells (n = >15 cells from two preparations per condition; P < 0.0001 with one-way ANOVA with Bonferroni's post test for comparison of fsk groups with I, H, C, and A). (H) Normalized iso concentration-response curve in freshly isolated (fresh) and cultured (cult) CAMPER_CM_ SAN cells. EC_50_ = 33.2 ± 1.2 nM for fresh cells and 35.7 ± 1.2 nM for cult cells (n = 10 cells per condition; P = 0.7861 with extra sum-of-squares F test). Significance (∗) was considered at P < 0.05. Data represent mean ± SEM.

In the next experimental series, cAMP signals were examined and compared between freshly dissociated and cultured adult mouse SAN cells from a cardiac-specific FRET-based cAMP reporter mouse. To generate this mouse, a recently developed conditional knockin mouse carrying the latest generation of the FRET-based Epac-mediated cAMP biosensor (e.g., CAMPER) (Muntean et al., 2018) was crossed with the genetically encoded driver line αMHC-Cre (CAMPER_CM_; Figures 2A and 2B). CAMPER_CM_ SAN cells also provide an independent functional test to validate the culturing method further. Freshly dissociated and cultured SAN cells from the adult CAMPER_CM_ mice exhibited strong YFP and CFP fluorescence (Figure 2C) and FRET ratio signals (Figure 2D). For measurement of FRET changes, (S)-nitro blebbistatin was not included in the imaging chamber solution. Application of a saturating concentration of the broad AC activator forskolin (10 μM) stimulated cAMP levels to similar levels in freshly dissociated and cultured CAMPER_CM_ SAN cells (Figures 2E and 2G, and Table S2). Treatment of cultured CAMPER_CM_ SAN cells with agonists known to trigger receptor-mediated cAMP synthesis (e.g., isoproterenol for β-adrenergic receptor [β-AR], histamine for histamine receptors, CGRP for CGRP receptors, and adenosine for adenosine receptors) also increased cAMP levels, although the magnitude of the responses was smaller when compared with forskolin responses, as expected (Figures 2F and 2G, and Table S2). Note that isoproterenol induced similar dose-dependent cAMP levels in freshly dissociated and cultured CAMPER_CM_ SAN cells (Figure 2H). These results suggest that the culturing method minimally alters, if any, cAMP signals and that it can be used to investigate cAMP dynamics in SAN cells.

**Figure 3:**
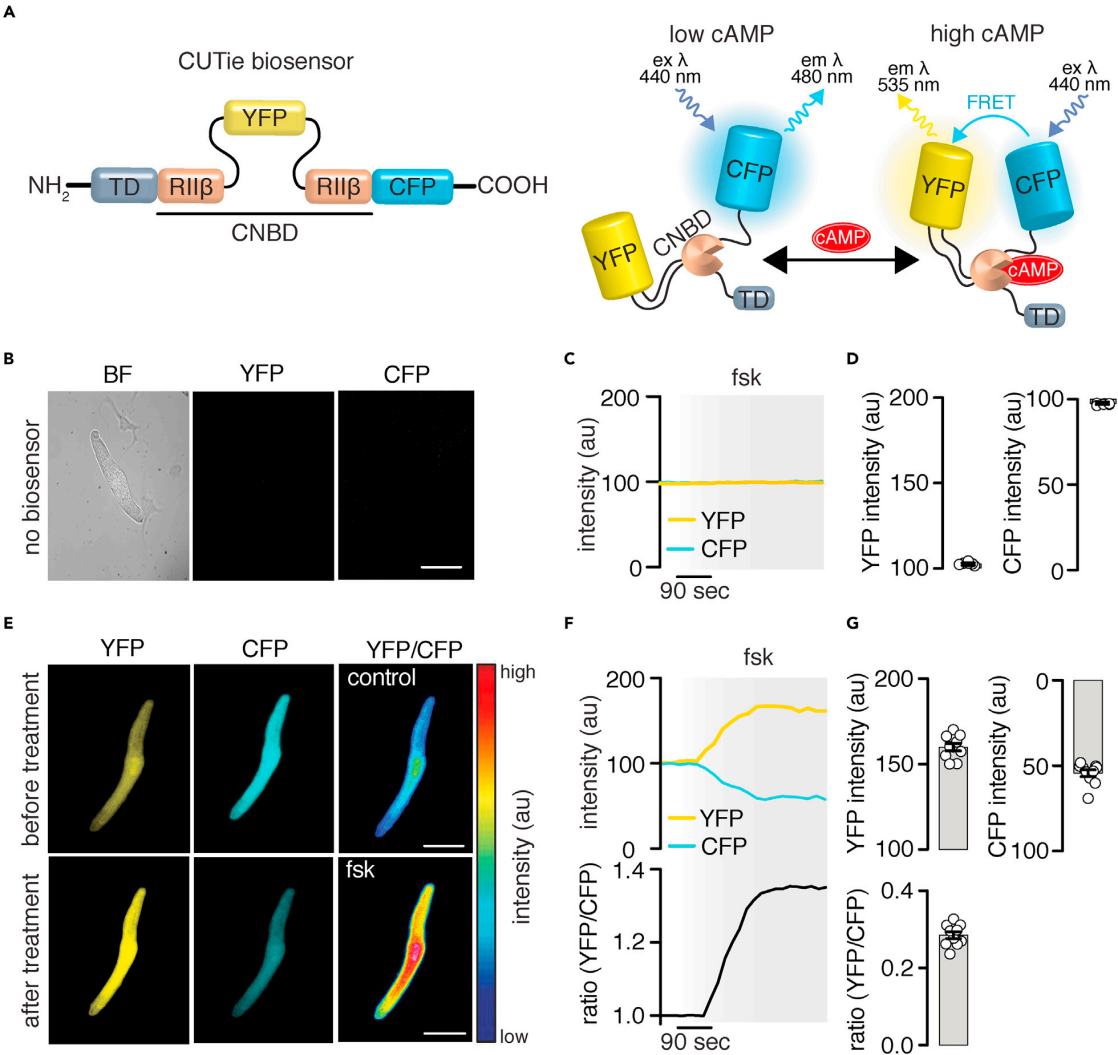
Expression of FRET-based sensors in adult mouse SAN cells (A) Diagram of the cytosolic FRET-based CUTie biosensor (Surdo et al., 2017). (B–D) Representative brightfield image of a wild-type adult mouse SAN cell. The cell was not infected with the CUTie biosensor as confirmed by the lack of YFP and CFP associated fluorescence in the accompanied images, as well as the lack of changes in YFP and CFP fluorescence intensity in response to 10 μM forskolin (fsk; C, D; n = 5 cells from two preparations). Scale bar, 10 μm. (E) Representative images of a wild-type adult mouse SAN cell showing robust YFP and CFP-associated fluorescence and pseudo-colored YFP/CFP ratio images before and after application of 10 μM fsk. Scale bar, 10 μm. (F) Time profile of YFP and CFP fluorescence, and YFP/CFP ratio changes in response to 10 μM fsk. (G) Scatterplot quantifying changes in YFP and CFP fluorescence, and YFP/CFP ratio after application of forskolin (n = 10 cells from three preparations). Data represent mean ± SEM.

To explore the utility of the adult mouse SAN cell culturing method for expression of exogenous proteins/probes, which can facilitate studies of cAMP dynamics, wild-type adult mouse SAN cells were infected with a new-generation FRET-based cAMP biosensor (e.g., cytosolic CUTie [Surdo et al., 2017], Figure 3A). Uninfected SAN cells cultured for 40 h in (S)-nitro blebbistatin showed no YFP or CFP fluorescence (Figure 3B), and application of forskolin had no effect on YFP/CFP intensity, as expected (Figures 3C and 3D). Adult mouse SAN cells infected with the cytosolic CUTie biosensor showed robust YFP and CFP fluorescence (Figure 3E). Forskolin induced an increase in YFP fluorescence and a decrease in CFP fluorescence that resulted in an augmentation in the FRET ratio signal (Figures 3F and 3G). These results are consistent with forskolin inducing an increase in cAMP levels in adult mouse SAN cells. Altogether, the results validate the culturing method and highlight its value (1) to express exogenous proteins/probes, (2) to examine receptor-mediated signaling, and (3) use of genetically modified mouse models to advance understanding of SAN cell regulation.

### Exploring cAMP pools and signaling networks in adult mouse SAN cells

**Figure 4:**
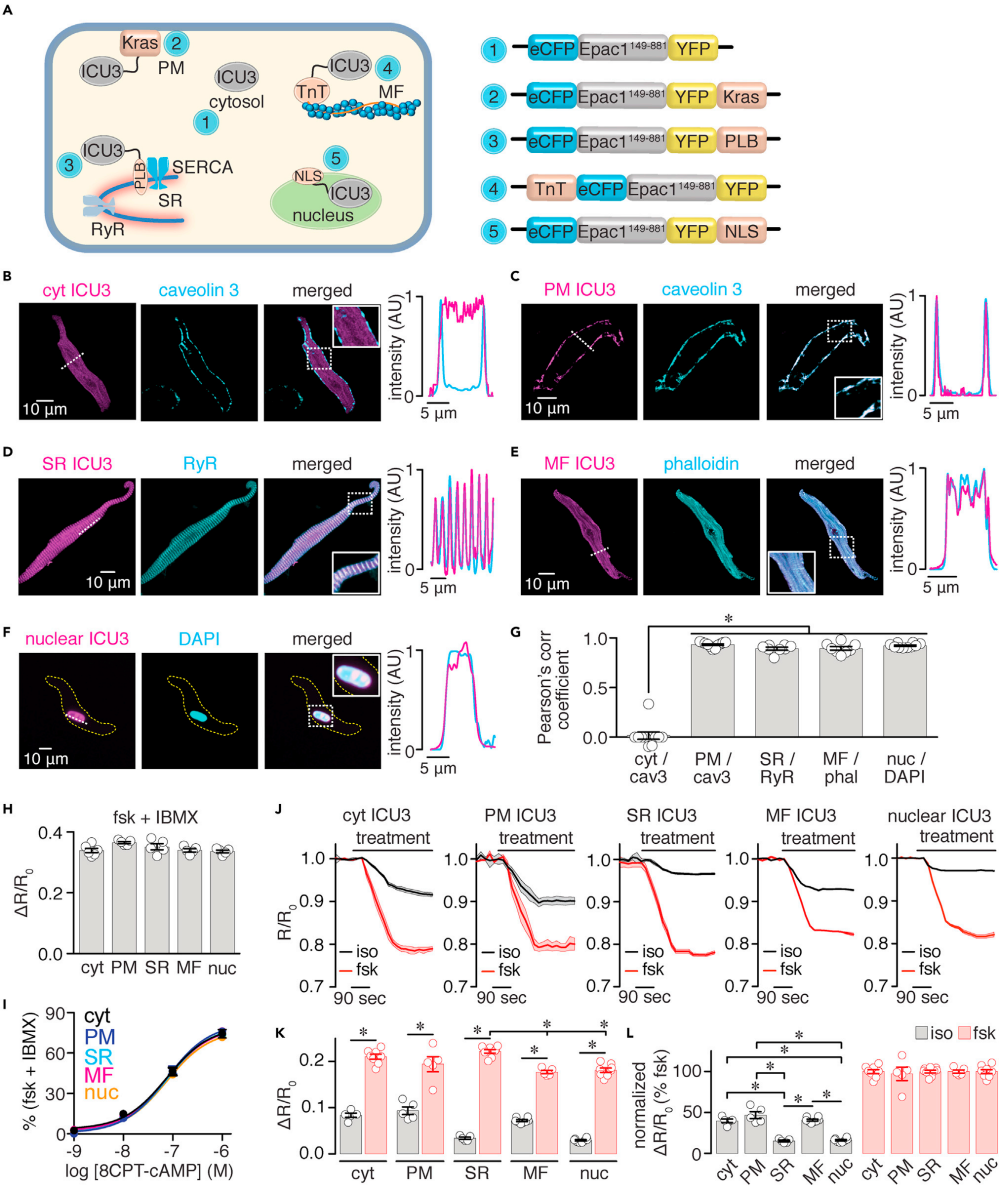
Discrete cAMP pools in adult mouse SAN cells (A) Diagrams highlighting localization and schematic representation of the Epac1-camps-based FRET biosensors (ICU3) in the cytosol (cyt; 1), plasma membrane (PM; 2), sarcoplasmic reticulum (SR; 3), myofilaments (MF; 4), and nucleus (nuc; 5). The ICU3 is linked to a Kras-derived sequence for PM localization, to a phospholamban (PLB)-derived sequence for SR localization, to a troponin T (TnT) for MF localization, and to a nuclear localization signal (NLS) sequence for nucleus localization. Exemplary super resolution images of adult wild-type mouse SAN cells expressing the indicated ICU3 biosensor in the cytosol (B), PM (C), SR (D), MF (E), and nucleus (F). The biosensor-associated fluorescence (YFP) is in magenta. Cells were immunostained with specific markers (in cyan) for the PM (caveolin 3), SR (ryanodine receptor 2 [RyR2]), MF (phalloidin; phal), and nucleus (DAPI). Merged images and corresponding line profile analysis (for dotted line) show high degree of overlap between the YFP fluorescence linked to the biosensor and the corresponding cellular marker in all cases, except in cells expressing the cytosolic sensor, as expected. Dotted squares highlight expanded regions in the solid squares. (G) Scatterplot of Pearson's correlation coefficient for cyt/cav3, PM/cav3, SR/RYR2, MF/phal, and nuc/DAPI (n > 8 SAN cells per condition). Kruskal-Wallis with Dunn's multiple comparisons test was used to test statistical differences in Pearson's correlation coefficient between non-target and targeted sensors. Scatterplot of the FRET ratio change in response to 10 μM forskolin (fsk) + 100 μM IBMX (H) and cAMP concentration-response curves (I) generated in HEK cells expressing the different ICU3 sensors (n > 5 cells per condition). For the cAMP concentration-response curves, cells expressing the different ICU3 sensors were exposed to increasing concentrations of the membrane-permeable cAMP analog 8CPT-cAMP. Kruskal-Wallis with Dunn's multiple comparisons test was used to compare fsk + IBMX responses, and the extra sum-of-squares F test was used to compare the cAMP EC_50_ response between sensors. (J) Average FRET ratio traces (mean, solid lines; SEM, shadow) in response to 100 nM isoproterenol (iso) or 10 μM fsk from adult wild-type mouse SAN cells expressing the cytosolic, PM, SR, MF, or nuclear ICU3 biosensors (n > 5 cells from three preparations per condition). Scatterplots of ΔR/R_0_ (K) and normalized (L) FRET responses after application of iso or fsk. Statistical differences were assessed with two-tailed Mann-Whitney test for comparisons between iso and fsk responses in (H) Statistical differences in fsk responses between the different biosensors in H were assessed with a Kruskal-Wallis with Dunn's multiple comparisons test. Statistical differences in normalized iso responses between the different groups were assessed using a one-way ANOVA with Tukey's multiple comparisons test. Significance (∗) was considered at P < 0.05. Exact p values are available in Table S3. Data represent mean ± SEM.

The next experimental series exploited the adult mouse SAN cell culturing method to examine cAMP pools and signaling networks in SAN cells, of which little is known. In the first set of experiments, adult mouse SAN cells were infected with the cytosolic Epac1-camp-based FRET biosensor (ICU3) or targeted sensors to the PM (PM-ICU3—Kras), SR (SR-ICU3—PLB), myofilaments (MF-ICU3—TnT), and nucleus (nuclear-ICU3—NLS) (Figure 4A) (Barbagallo et al., 2016; DiPilato and Zhang, 2009). These biosensors can report cAMP level changes within a specific compartment (Barbagallo et al., 2016; DiPilato and Zhang, 2009; Reddy et al., 2018; Zaccolo et al., 2021). Super-resolution imaging of cells infected with the different biosensors shows an anticipated expression pattern for the targeted regions, as confirmed by line profile analysis of the biosensors with specific markers for each region (Figures 4B–4F) and Pearson's correlation coefficients (Figure 4G and Table S3) compared with the cytosolic sensor. The in-cell ICU3 sensors showed similar maximal ratio amplitude in response to fsk and 3-Isobutyl-1-methylxanthine (IBMX) (Figure 4H and Table S3) and cAMP concentration-response curves (Figure 4I and Table S3). These results suggest that the ICU3 sensors used in this study have comparable dynamic range and cAMP sensitivity.

SAN cells expressing the ICU3 sensors showed that application of the β-AR agonist isoproterenol increased cAMP levels in all compartments, and this was significantly amplified by a saturating concentration of forskolin (10 μM; Figures 4J and 4K, and Table S3). Forskolin response was of comparable amplitude in most compartments with slight but statistical reductions between the SR and MF and SR and nucleus (Figure 4K and Table S3). Normalization of the isoproterenol response to the average magnitude of the forskolin response suggests distinct cAMP pools in SAN cells, with the cytosol (40%), PM (47%), and MF regions (41%) showing larger cAMP levels compared with SR (15%) and nuclear (16%) compartments (Figure 4L and Table S3). These results suggest that the 100 nM isoproterenol stimulation causes distinctive receptor-mediated cAMP levels in different subcellular regions, suggesting that this second messenger may be compartmentalized in adult mouse SAN cells.

**Figure 5:**
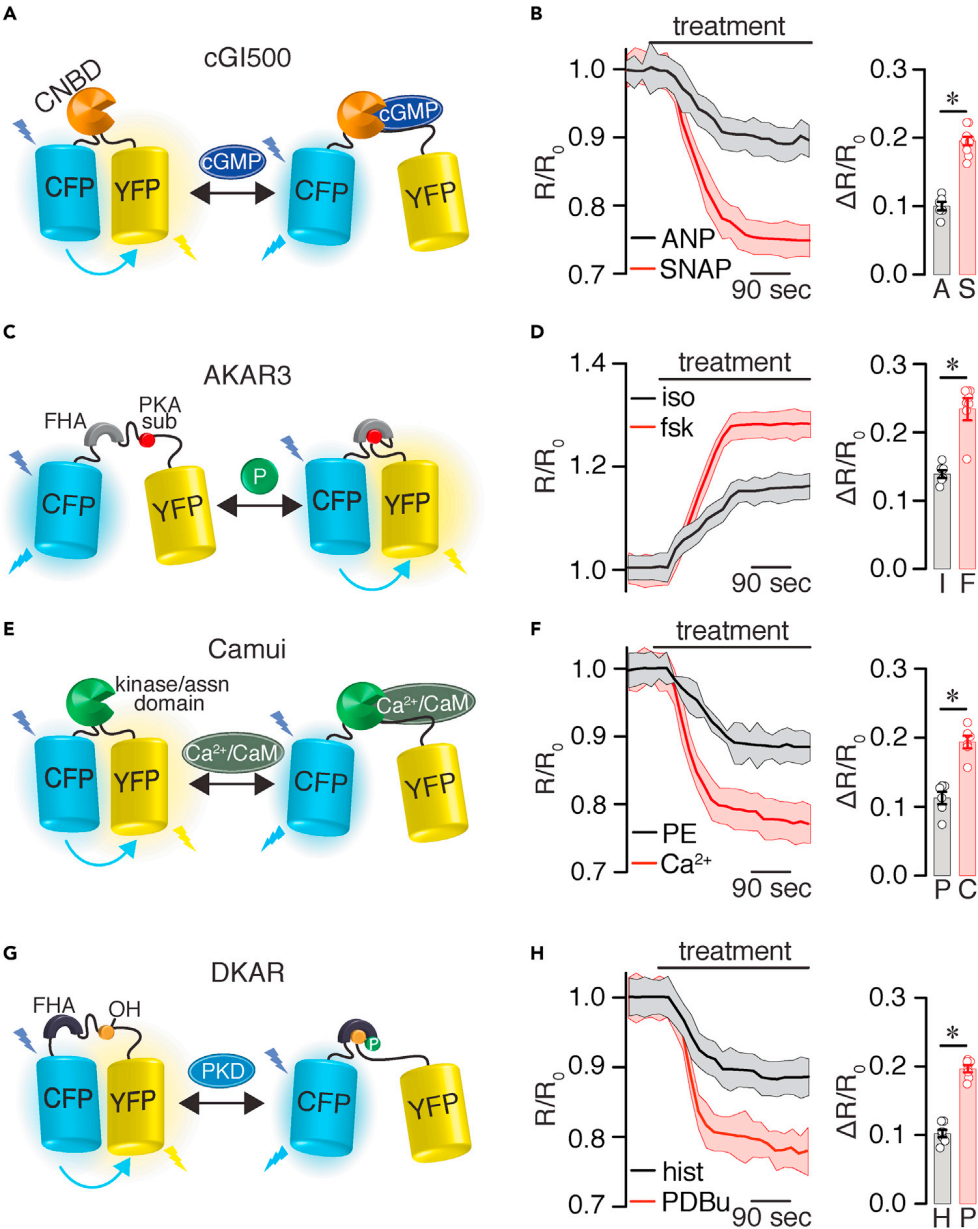
Detection of different signaling networks in adult mouse SAN cells (A) Schematic diagram of cGMP reporter cG1500. (B) Average FRET ratio traces (mean, solid lines; SEM, shadow) in response to 100 μM ANP or 100 μM SNAP and scatterplots of ΔR/R_0_ treatment effect (n = >6 cells from three preparations per condition). A, ANP; S, SNAP. (C) Schematic diagram of the PKA activity reporter AKAR3. (D) Average FRET ratio traces (mean, solid lines; SEM, shadow) in response to 100 nM isoproterenol or 10 μM forskolin and scatterplots of ΔR/R_0_ treatment effect (n = >6 cells from three preparations per condition). I, isoproterenol; F, forskolin. (E) Schematic diagram of the CaMKII activity reporter Camui. (F) Average FRET ratio traces (mean, solid lines; SEM, shadow) in response to 1 μM PE or 200 μM calcium and scatterplots of ΔR/R_0_ treatment effect (n = >6 cells from three preparations per condition). P, PE; C, calcium. (G) Schematic diagram of the PKD activity reporter DKAR. (F) Average FRET ratio traces (mean, solid lines; SEM = shadow) in response to 10 μM histamine or 500 nM PDBu and scatterplots of ΔR/R_0_ treatment effect (n = >6 cells from three preparations per condition). Statistical differences were assessed using a two-tailed Mann-Whitney test. Significance (∗) was considered at P < 0.05. Exact p values are available in Table S4. Data represent mean ± SEM.

The second set of experiments explored the utility of the adult mouse SAN cell culturing method to study other signaling networks. For this, adult mouse SAN cells were infected with different well-validated biosensors, including the cGi500 for cGMP signaling (Russwurm et al., 2007; West et al., 2019), AKAR3 for PKA activity (Allen and Zhang, 2006; Liu et al., 2012), Camui for CaMKII activity (Erickson et al., 2011; Takao et al., 2005), and DKAR for PKD activity (Bossuyt et al., 2011; Kunkel et al., 2007). Application of receptor-mediated stimuli to SAN cells, such as atrial natriuretic peptide (ANP), isoproterenol, phenylephrine (PE), and histamine, triggered subtle but significant changes in FRET ratio, which is consistent with increased cGMP production and enhanced PKA, CaMKII, and PKD activity, respectively (Figures 5A–5H and Table S4). These responses were further amplified by the addition of S-nitroso-N-acetylpenicillamine (SNAP), forskolin, calcium, and phorbol 12,13-dibutyrate (PDBu), which are known to stimulate global cGMP production and global activity of PKA, CaMKII, and PKD, respectively (Figures 5A–5H and Table S4). These results suggest that a range of responses in cGMP production and activity of PKA, CaMKII, and PKD can be detected in SAN cells. Moreover, the results highlight that the culturing method can be used to study different signaling networks in adult mouse SAN cells.

### Interrogating SAN cells signaling networks in disease

**Figure 6:**
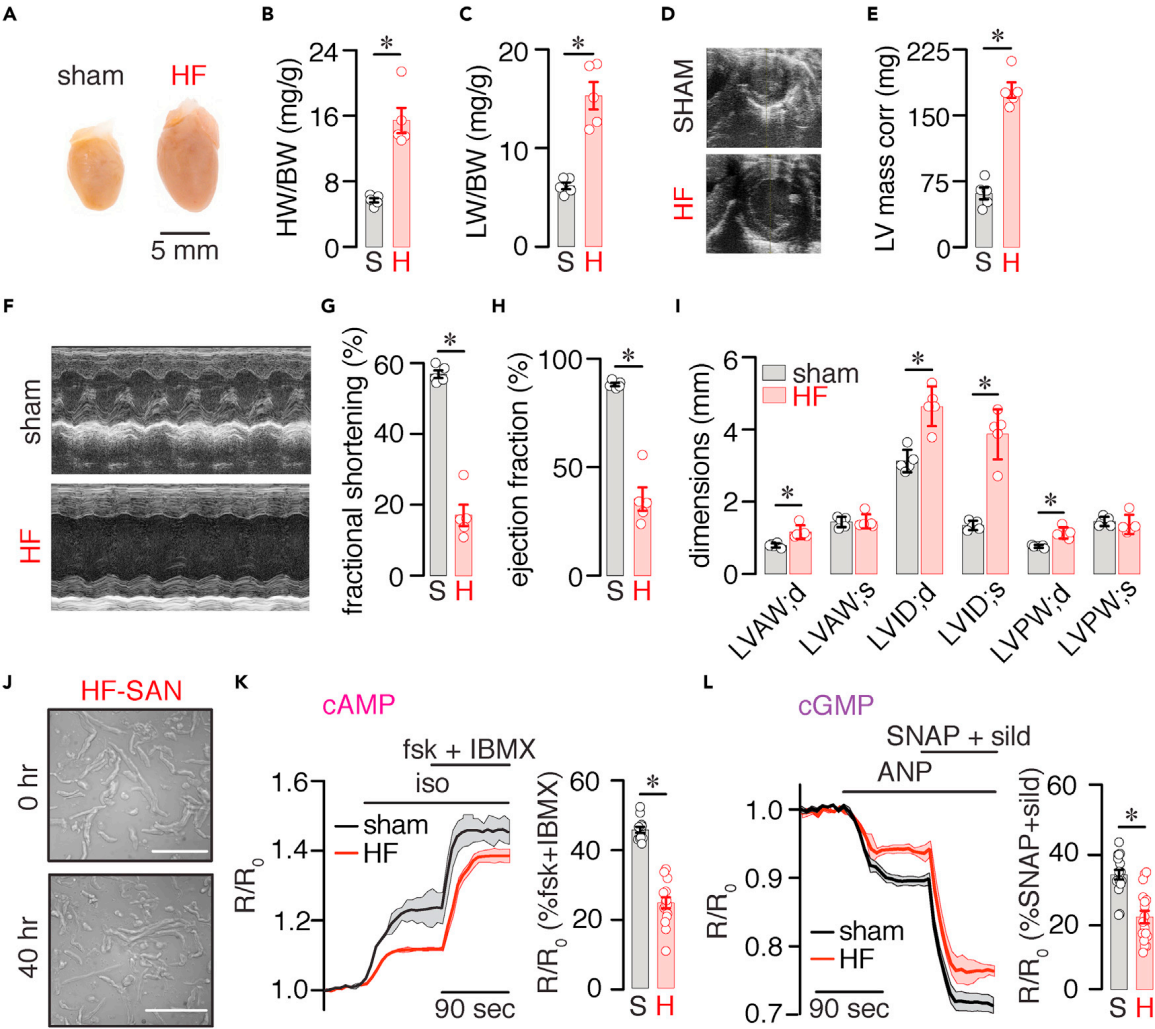
cAMP and cGMP signaling in adult mouse SAN cells during HF (A) Representative whole heart images taken at 8 weeks after sham and TAC (HF) surgery. Scatterplot of (B) heart weight to body weight (HW/BW) ratio (P = 0.0004 with unpaired t test) and (C) lung weight to body weight (LW/BW) ratio (P = 0.0214 with unpaired t test) from sham and HF mice (n = 5 hearts per condition). (D) Representative images of sham and HF hearts at the parasternal short axis. (E) Summary scatterplot data of corrected left ventricular (LV) mass in sham and HF mice (P = 0.0005 with unpaired t test; n = 5 hearts per condition). (F) Representative M-mode echocardiographic images for sham and HF mice. Scatterplot data from echocardiography for (G) fractional shortening (P = 0.0009 with unpaired t test), (H) ejection fraction (P = 0.0029 with unpaired t test), and cardiac dimensions (I) from sham and HF mice (n = 5 hearts per condition). LVAW;d, left ventricular arterial wall—diastole (P = 0.0029 with t test); LVAW;s, left ventricular arterial wall—systole (P = 0.8560 with t test); LVID;d, left ventricular interior diameter—diastole (P = 0.0007 with t test); LVID;s, left ventricular interior diameter—systole (P = 0.00004 with t test); LVPW;d, left ventricular posterior wall—diastole (P = 0.0008 with t test); LVPW;s, left ventricular posterior wall—systole (P = 0.5384 with t test). (J) Brightfield images of adult wild-type mouse sham and HF SAN cells at 0 and 40 h in culture. Scale bar, 10 mm. (K) Average FRET ratio traces (mean, solid lines; SEM, shadow) in response to 100 nM isoproterenol and 10 μM forskolin + 100 μM IBMX and scatterplots of R/R_0_ treatment effect (n = >10 cells from three preparations per condition) in sham (S) and HF (H) SAN cells (Mann-Whitney test). (L) Average FRET ratio traces (mean, solid lines; SEM, shadow) in response to 100 μM ANP and 100 μM SNAP + 1 μM sildenafil and scatterplots of maximum treatment effect (n = >10 cells from three preparations per condition) in sham (S) and HF (H) SAN cells (Mann-Whitney test). Significance was considered at P < 0.05. Exact p values are available in Table S5. Data represent mean ± SEM.

SAN cell dysfunction is a well-described manifestation in patients with heart failure (HF) and HF animal models (Sanders et al., 2004; Verkerk et al., 2003). Thus, the final set of experiments determined if the adult mouse SAN cell culturing method can be employed to examine signaling networks in HF SAN cells. To do this, a well-established pressure overload model of HF was used (e.g., transverse aortic constriction, TAC) (Sirish et al., 2013). HF mice exhibited cardiac hypertrophy and dilatation (Figures 6A and 6B), with evidence of pulmonary congestion (Figure 6C), increased left ventricular mass (Figures 6D and 6E), depressed systolic function (Figures 6F–6H), and several alterations in cardiac dimensions (Figure 6I) compared with sham. The results provide evidence that TAC mice developed impaired cardiac function and structural remodeling consistent with HF.

With the HF model in place, SAN cells from sham and HF mice were isolated and cultured in (S)-nitro blebbistatin as described in the STAR Methods section. HF SAN cells displayed a slight decrease in cell length at both time points with no changes in cell shape at 40 h compared with sham cells (Figure 6J and Table S5). Cell length and shape were still significantly higher in HF SAN cells cultured in (S)-nitro blebbistatin than in wild-type cells maintained in M1018 media alone. Moreover, cell length and shape were similar between HF SAN cells at 0 and 40 h (Table S5).

Sham and HF SAN cells were then infected with either the cytosolic CUTie and cGi500 FRET biosensors to examine cAMP and cGMP signaling, respectively. Sham cells showed increased cAMP production in response to isoproterenol, which was further amplified by applying forskolin and IBMX to elicit maximal cAMP (Figure 6K). Likewise, ANP augmentation of cGMP levels was further increased by SNAP and the phosphodiesterase inhibitor sildenafil to promote maximal cGMP levels (Figure 6L). However, the isoproterenol and ANP responses were significantly reduced in HF SAN cells (Figures 6K and 6L and Table S5). These results suggest that cAMP and cGMP production is impaired in SAN cells during HF. In addition, the data highlight the utility of the SAN cell culturing method to examine signaling networks in adult mouse SAN cells during pathological conditions.

## Discussion

The present study describes a method for culturing adult mouse SAN cells based on treatment with the contraction uncoupler (S)-nitro blebbistatin. Advantages of this method are that it (1) helps retain SAN cell morphology in a larger number of cells than other methods (e.g., BDM), (2) preserves protein distribution and electrical and signaling properties of cultured cells for at least 40 h, (3) can be used to express functional exogenous proteins and probes, and (4) is easily adaptable for the use of SAN cells from genetically modified mice and disease-state mouse models. By exploiting this culturing method, the following important findings were made in adult mouse SAN cells: (1) discovery of β-AR-mediated discrete cAMP pools consistent with compartmentalization; (2) detection and examination of cGMP production and activity of PKA, CaMKII, and PKD, thus facilitating the study of signaling networks; and (3) reduction in cAMP and cGMP levels that could contribute to SAN dysfunction during HF. These results highlight the significance of the culturing method and how it may open opportunities to gain critical insights into the regulation of SAN cells in health and disease.

Contraction uncouplers, such as BDM and blebbistatin, have been used to preserve the morphology and sustained cultured cardiac cells for functional studies (Kabaeva et al., 2008; Kirschner Peretz et al., 2017; Kivisto et al., 1995; Liu et al., 1996; Muramatsu et al., 1996; Reddy et al., 2018; Segal et al., 2019; St Clair et al., 2015; Yang et al., 2012). Initial efforts to culture adult mouse SAN cells by St Clair et al. (2015) showed that BDM could help preserve cell morphology, AP waveform, and electrical properties. However, it is not clear whether the authors compared the cell morphology of a large population of cells within the same field of view, which is important to assess the effectiveness of the culturing method. Note that, in the present study, one can still spot a very small number of cultured cells in M1018 and BDM with cell length and shape comparable with cell properties at 0 h in culture (see Figure 1). St Clair et al. (2015) also reported that treating mouse SAN cells with a relatively high concentration of blebbistatin was ineffective in maintaining cell morphology and viability. However, the data were not shown, which made difficult an appropriate comparison between studies. Conversely, recent studies have suggested that blebbistatin and its non-fluorescent derivative (S)-nitro blebbistatin (Kepiro et al., 2014) may be more effective at improving survival and preserving the morphology of cultured rabbit SAN cells and ventricular myocytes (Kirschner Peretz et al., 2020; Reddy et al., 2018; Segal et al., 2019). Consistent with this, by analyzing a population of SAN cells within the same field of view, the present study found that blebbistatin and (S)-nitro blebbistatin help maintain cell morphology in a significantly larger number of cultured cells compared with treatments with M1018 or BDM (Figure 1).

Experimental and *in silico* modeling suggested that the benefits of blebbistatin may stem from its ability to maintain cellular energy and biochemical properties (Segal et al., 2019). Consistent with this, the present study shows that treating adult mouse SAN cells with (S)-nitro blebbistatin preserved the AP waveform and cAMP signaling properties (e.g., dynamic range and sensitivity) in cultured adult mouse SAN cells compared with freshly dissociated cells (Figures 1 and 2). These results suggest that the biochemical and electrical properties as well as signaling pathways are likely preserved in (S)-nitro blebbistatin-cultured cells. The difference in results of culturing with blebbistatin between the present study and that of St Clair et al. (2015) may stem from their use of high blebbistatin concentrations (25 versus 6.5 μM here). Since blebbistatin breakdown may lead to the generation of reactive oxygen species (Kolega, 2004; Sakamoto et al., 2005), high blebbistatin concentrations could be detrimental for mouse SAN cell viability and function. Intriguingly, the Yaniv lab also considered the use of BDM and blebbistatin to culture adult SAN cells from rabbits (Segal et al., 2019). For this, they used 25 μM blebbistatin, the same blebbistatin concentration used by the Proenza lab to culture mouse SAN cells (St Clair et al., 2015). The Yaniv lab found that 25 μM blebbistatin was more effective for culturing rabbit SAN cells than BDM (Segal et al., 2019). Perhaps rabbit SAN cells are more resilient to the potential side effects of 25 μM blebbistatin than mouse SAN cells. However, Yaniv and colleagues also reported that at least 13% of the rabbit SAN cells in their culturing method with 25 μM blebbistatin lost their spindle-shaped morphology and developed projections (Segal et al., 2019). This morphological change was not observed in mouse SAN cells cultured with 6.5 μM blebbistatin or 20 μM (S)-nitro blebbistatin. These observations suggest that the use of blebbistatin/(S)-nitro blebbistatin at the indicated concentration in this study may be more effective for culturing of adult mouse SAN cells.

A potential complication with blebbistatin is that it may increase cell autofluorescence (Reddy et al., 2018), which may interfere with fluorescent probes. This issue can be resolved by culturing cells in non-fluorescent (S)-nitro blebbistatin (Kepiro et al., 2014). Indeed, the use of (S)-nitro blebbistatin was found to reduce ventricular myocyte autofluorescence compared with blebbistatin, and this was essential to record FRET signals in these cells (Reddy et al., 2018). In the current study, (S)-nitro blebbistatin in the culture media did not significantly impact FRET signals, as reflected by cAMP levels of similar magnitude in freshly dissociated and cultured SAN cells in the culture media response to forskolin (Figures 2 and 4). Thus, blebbistatin/(S)-nitro blebbistatin are useful compounds for culturing adult mouse SAN cells that retain morphological, electrical, and signaling properties.

The applicability of the culturing method was underscored by demonstrating that adult mouse SAN cells can be infected with exogenous proteins/probes for functional studies. The approach can be extended to use SAN cells from genetically modified mice and models of disease. Accordingly, adult mouse SAN cells infected with the FRET-based cAMP biosensors show robust CFP and YFP fluorescence signals (Figures 2 and 3). These signals changed in intensity, as predicted for the FRET sensors, in response to stimuli that increase cAMP levels (Figure 3). Notably, the culturing method did not seem to affect the cAMP biosensor sensitivity as the FRET ratios were similar in fresh and cultured SAN cells (Figures 2 and 4), and the biosensors showed expected subcellular localization for the targeted region (Figure 4). The culturing method was also effective at preserving cell morphology and survival of HF SAN cells (Figure 6 and Table S5), which facilitated infection of these cells with FRET-based biosensors for the study of signaling networks in HF conditions. Thus, the adult mouse SAN cell culturing method opens many avenues to interrogate the regulatory mechanisms of these cells.

It is anticipated that the culturing method can be applied to express many biosensors to study lipids, pH, and glucose metabolism, for example. It can be used to express genetically encoded calcium indicators for the independent or combined examination of calcium dynamics and signaling networks. In addition, one can apply the method for knockout/down, overexpression, or rescue of proteins of interest, as well as for the culture of adult SAN cells from higher-order species, including rabbits and humans, to perform experiments similar to those described here. In support of the latter possibility, a recent study found that rabbit SAN cells cultured in blebbistatin-containing media maintained their AP firing rate and calcium signaling properties compared with fresh cells (Segal et al., 2019). Future studies are required to test if this approach with (S)-nitro blebbistatin facilitates the infection of SAN cells from higher-order species with FRET-based biosensors to study signaling networks.

A major goal in developing an adult mouse SAN cell culturing method is to gain insight into mechanisms regulating SAN cell function. Compartmentalization of cAMP signaling is a well-established regulatory mechanism in ventricular myocytes in health and disease (Zaccolo et al., 2021). Recent experimental and *in silico* studies have suggested that cAMP signaling may also be compartmentalized in SAN cells (Behar et al., 2016; Vinogradova et al., 2018a, 2018b; Yaniv et al., 2015a), but whether this is the case remained unclear until now. By expressing cAMP reporters to specific subcellular regions, this study provides evidence of discrete cAMP pools, consistent with cAMP compartmentalization in adult mouse SAN cells (Figure 4). Accordingly, the application of the β-AR agonist isoproterenol stimulated a larger increase in cAMP levels in the cytosol and at the PM and MF than in the SR and nuclear compartments of adult mouse SAN cells. Compartmentalization of cAMP signaling may be essential for fine-tuning the activity of coupled-clock proteins and pacemaking activities in SAN cells (Behar et al., 2016; Vinogradova et al., 2018a, 2018b; Yaniv et al., 2015a). Another intriguing aspect highlighted by the data is that cAMP pools in SAN cells seem fundamentally distinct from ventricular myocytes (Surdo et al., 2017). Accordingly, in ventricular myocytes the PM and SR regions experience similar levels of β-AR-induced cAMP, with the MF compartment showing different cAMP amplitudes and kinetics (Surdo et al., 2017). Conversely, SAN cells show similar cAMP levels in response to β-AR stimulation in the PM and MF compartments with reduced cAMP levels in the SR region (Figure 4). Therefore, data here provide a strong rationale for future studies to directly test mechanistic underpinnings of cAMP compartmentalization in SAN cells and how they differ from those in ventricular myocytes.

Another important insight gained is the dynamics of different signaling networks known to regulate SAN function in health and disease (Behar and Yaniv, 2016; DiFrancesco and Borer, 2007; Han et al., 1995; Liao et al., 2010; Musialek et al., 1997; Peters et al., 2020; Shimizu et al., 2002; Vinogradova and Lakatta, 2009; Vinogradova et al., 2000, 2018b; Wood and Bossuyt, 2017; Wu and Anderson, 2014; Wu et al., 2009; Xie et al., 2015; Yaniv et al., 2013, 2015a, 2015b). By infecting adult mouse SAN cells with FRET-based biosensors to measure cAMP and cGMP levels or PKA, CaMKII, and PKD activity, one can track the magnitude of the response of these signaling networks during physiological and pathological conditions (Figures 4, 5 and 6). Ligands that are known to act via receptor-mediated signaling (e.g., ANP, isoproterenol, PE, and histamine) were found to produce smaller FRET ratio changes than stimuli known to induce total or global effects (e.g., SNAP, forskolin, calcium, and PDBu) (Figures 4 and 5). These results indicate that the extent of second messenger production and activity of different signaling networks can be distinguished with the approach employed here. Understanding the dynamic range of responses in the various signaling networks is important to link their effects to functional outcomes. Consistent with this, it has been shown that the kinetics of PKA activity matches that of the spontaneous SAN cell AP firing rate in response to changes in β-AR activity (Behar et al., 2016; Yaniv et al., 2015a). It is also likely that CaMKII dynamics are tightly linked to SAN automaticity as this kinase phosphorylates many coupled-clock proteins (Vinogradova et al., 2000; Wu and Anderson, 2014; Wu et al., 2009; Xie et al., 2015; Yaniv et al., 2013). Whether similar graded patterns of cAMP, cGMP, and PKD signaling regulate SAN pacemaking activity remains unclear.

Data in this study also showed that HF SAN cells displayed reduced receptor-mediated cytosolic cAMP and cGMP levels in response to isoproterenol and ANP, respectively, compared with sham (Figure 6). The reduction in SAN cell cAMP and cGMP signaling may contribute to SAN dysfunction during HF by altering the second messenger activity via negative feedback regulation between them, the activity of coupled-clock proteins, or the function of other signaling pathways (MacDonald et al., 2020). Thus, the culturing method described here for adult mouse SAN cells may be exploited to gain insight into signaling networks properties and how they may control SAN function in health and disease.

In summary, this study describes a robust, efficient, and easy-to-implement adult mouse SAN cell culturing method. By leveraging this method, evidence describes discrete cAMP pools, the detection of several signaling network dynamics, and alterations in receptor-mediated cAMP and cGMP levels during HF in adult mouse SAN cells. The culturing method and results given here open opportunities to gain fundamental insights into mechanisms regulating SAN function in health and disease.

### Limitations of the study

One limitation in this study is the use of mouse SAN cells, which have electrical, calcium, and likely signaling network properties that may differ from those found in human SAN cells (MacDonald et al., 2020). Nonetheless, mouse SAN cells provide an accessible, highly manipulable, distinguishable, and robust system to unmask and examine many SAN cell properties, including signaling networks that may control SAN function (St Clair et al., 2015). Because the culturing method described here is relatively simple, it can be easily adapted for culturing SAN cells from higher-order species, where the translation of the knowledge gained from mouse SAN cells can be tested. Although cAMP signaling seems to be preserved in freshly dissociated and cultured adult mouse SAN cells (based on cAMP mouse reporter), long-term culture of higher-order species SAN cells with blebbistatin/(S)-nitro blebbistatin may alter cell properties. This concern, however, is ameliorated by a recent report indicating that electrical and calcium properties are similar in fresh and cultured rabbit SAN cells (Segal et al., 2019). SAN cell heterogeneity, both within a similar population of cells and across the SAN tissue (Boyett et al., 2000; Brennan et al., 2020; Grainger et al., 2021), could be further exacerbated by the culturing conditions. Given that blebbistatin/(S)-nitro blebbistatin significantly improve cell survival, investigators will be able to acquire data with high statistical power by recording from many more cells. At the moment, it cannot be ruled out that overexpression of FRET biosensors may affect the integrity of the signaling networks in SAN cells. Despite these potential limitations, the adult mouse SAN cell culturing method has tremendous potential to unmask mechanisms regulating SAN function.

## STAR★Methods

### Key resources table

**Table:** 

REAGENT or RESOURCE	SOURCE	IDENTIFIER
**Antibodies**		

Rat monoclonal anti-HCN4	Abcam	Cat# ab32675; RRID: AB_732770
Goat polyclonal anti-AC_VI_	Santa Cruz	Cat# sc68138; RRID: AB_1563121
Rabbit polyclonal anti-caveolin 3	Thermo Fisher	Cat# PA1-066; RRID: AB_2072446
Rabbit polyclonal anti RyR2	Alomone	Cat# ARR-002; RRID: AB_2040184
Alexa Fluor 555-conjugated donkey anti-rabbit	Thermo Fisher	Cat# A-31572; RRID: AB_162543
Alexa Fluor 555-conjugated donkey anti-rat	Abcam	Cat# ab150150
Alexa Fluor 647-conjugated donkey anti-goat	Abcam	Cat# ab150135

**Bacterial and virus strain**s		

ICU3	Barbagallo et al. (2016), DiPilato and Zhang (2009)	N/A
PM-ICU3	Barbagallo et al. (2016), DiPilato and Zhang (2009)	N/A
SR-ICU3	Barbagallo et al. (2016), DiPilato and Zhang (2009)	N/A
TnT-ICU3	Barbagallo et al. (2016), DiPilato and Zhang (2009)	N/A
NLS-ICU3	Barbagallo et al. (2016), DiPilato and Zhang (2009)	N/A
CUTie	Zaccolo et al. (2021)	N/A
AKAR3	Allen and Zhang (2006)	N/A
cGI500	Russwurm et al. (2007)	N/A
Camui	Takao et al., (2005)	N/A
DKAR	Kunkel et al. (2007)	N/A

**Chemicals, peptides, and recombinant proteins**		

Laminin	Life technology	Cat# 23017015
Collagenase B	Sigma Aldrich	Cat# 11088815001
Elastase	Sigma Aldrich	Cat# 45124
Protease type	Sigma Aldrich	Cat# P5147
Media M1018	Sigma Aldrich	Cat# M1018
2,3-Butanedione 2-monoxime (BDM)	Sigma Aldrich	Cat# B0753; CAS: 57-71-6
Blebbistatin	Cayman	Cat# 13013; CAS: 856925-71-8
(S)-nitroblebbistatin	Cayman	Cat# 13891; CAS: 856925-75-2
Forskolin	Sigma-Aldrich	Cat# F6886; CAS: 66575-29-9
Isoproterenol	Sigma-Aldrich	Cat# I2760; CAS: 54750-10-6
Histamine	Sigma-Aldrich	Cat# H7125; CAS: 51-45-6
Calcitonin gene-related peptide (CGRP)	Sigma-Aldrich	Cat# C0167; CAS: 90954-53-3
Atrial natriuretic peptide (ANP)	Sigma-Aldrich	Cat# A1663; CAS: 91917-63-4
S-nitroso-N-acetylpenicillamine (SNAP)	Sigma-Aldrich	Cat# N3398; CAS: 67776-06-1
Phenylephrine (PE)	Sigma-Aldrich	Cat# P6126; CAS: 6176-7
phorbol 12,13-dibutyrate (PDBu)	CalBiochem	Cat# 524390; CAS: 37558-16-0
Sildenafil	Sigma-Aldrich	Cat# SML3033; CAS: 171599-83-0
Alexa Fluor 647 Phalloidin	Thermo Fisher	Cat# A22287
ProLong Diamond Antifade Mountant	Thermo Fisher	Cat# P36970
8CPT-cAMP	Abcam	ab120424

**Experimental models: Cell Lines**		

HEK cells	Sigma-Aldrich	Cat #: ECACC 96121229; RRID:CVCL_2737

**Experimental models: Organisms/strains**		

Mouse: C57BL/6J	The Jackson Laboratory	JAX 000664
Mouse: C57BL/6-*Gt(ROSA)26Sor*^*tm1(CAG-ECFP∗/Rapgef3/Venus∗)Kama*^/J; (CAMPER floxed)	The Jackson Laboratory	JAX 032205
Mouse: B6.FVB-Tg(Myh6-cre)218Mds/J; (cardiac-specific alpha myosin-heavy chain (*Myh6*) Cre mouse)	The Jackson Laboratory	JAX 011038
Mouse: cardiac-specific CAMPER mice (CAMPER_CM_)	This paper	N/A

**Software and algorithms**		

Metafluor	Molecular Devices	https://www.moleculardevices.com/sites/default/files/en/assets/brochures/dd/img/metafluor-fluorescence-ratio-imaging-software.pdf; RRID:SCR_014294
Graph Pad Prism	GraphPad Software	https://www.graphpad.com/; RRID:SCR_002798
VisualSonics Vevo 2100	FUJIFILM VisualSonics	https://www.visualsonics.com/
ImageJ	National Institute of Health	https://imagej.nih.gov/ij/; RRID:SCR_003070
pClamp10	Molecular Devices	https://www.moleculardevices.com/products/axon-patch-clamp-system/acquisition-and-analysis-software/pclamp-software-suite#gref; RRID:SCR_011323
Imaris	Oxford Instruments	https://imaris.oxinst.com; RRID:SCR_007370

### Resource availability

#### Lead contact

Further information and requests for resources and reagents should be directed to and will be fulfilled by the lead contact, Dr. Manuel F. Navedo (mfnavedo@ucdavis.edu).

#### Material availability

• Viruses for the different FRET-based biosensors are available from the lead contact upon request.
• The CAMPER floxed mouse (stock No: 032205) and the *Myh6* Cre mouse (stock No: 011038) can be obtained from The Jackson Laboratories. The CAMPER_CM_ mouse line may be available from the lead contact upon request and availability of mice.
• This study did not generate new unique reagents or standardized datatypes.

### Experimental models and subject details

#### Animals

Male wild type mice 8-10 weeks old in C57Bl6/J background (The Jackson Laboratory, Sacramento, CA) were used for this study. Number of mice used for each experimental series are included in the Figure legends. Mice were maintained in 12 hours light/12 hours dark cycle environment with an *ad libitum* food supply. All studies conform with the US National Institutes of Health Guide for the Care and Use of Laboratory Animals (NIH publication No. 85-23, revised 1985) and was performed in accordance with the protocols and guidelines approved by the Animal Care and Use Committee of the University of California, Davis.

#### Generation of cardiac specific CAMPER (CAMPER_CM_) mouse

The CAMPER floxed mouse (stock No: 032205) and the cardiac-specific alpha myosin-heavy chain (*Myh6*) Cre mouse (stock No: 011038) were obtained from The Jackson Laboratories. The CAMPER mouse was generated by Kirill Martemyanov of The Scripps Research Institute (Muntean et al., 2018). To generate the cardiac-specific CAMPER mice (CAMPER_CM_), CAMPER floxed and *Myh6* Cre mice were crossed to obtained CAMPER^+/+^; Cre^+/+^ breeding pairs that could be used to maintain the CAMPER_CM_ colony. No apparent signs of development deficiencies were observed, although this has not been rigorously examined. Mice were genotyped before use. Male and female CAMPER_CM_ mice 8-10 weeks old were used for experiments.

#### Adult mouse SAN cell isolation

Adult mouse SAN cells from male wild type mice were isolated following established procedures (Fenske et al., 2016; Mangoni and Nargeot, 2001; Sharpe et al., 2016; Vinogradova et al., 2000). Mice were anesthetized by intraperitoneal injection of 80 mg/kg of ketamine and 5 mg/kg of xylazine. The heart was excised and placed into Tyrode's solution (35°C) containing (in mM): 140 NaCl, 5.0 HEPES, 5.5 glucose, 5.4 KCl, 1.8 CaCl_2_, and 1.0 MgCl_2_ (pH 7.4). The SAN tissue was dissected based on the landmarks defined by the orifice of superior vena cava, crista terminalis, and atrial septum under a dissection microscope (Zhang et al., 2002). SAN tissue was digested in low calcium solution containing (in mM): 140 NaCl, 5.0 HEPES, 5.5 Glucose, 5.4 KCl, 0.2 CaCl_2_ and 0.5 MgCl_2_, 1.2 KH_2_PO_4_, 50 taurine, pH 6.9, with collagenase B (0.54U/mL, Sigma-Aldrich, St Louis, MO), elastase (18.9 U/mL, Sigma-Aldrich) and protease type XIV (1.79 U/mL, Sigma-Aldrich) for 30 mins at 37°C in the water bath. After digestion, the SAN tissue was centrifuged at 200g for 2 mins at 4°C in order to stop the digestion. After centrifuging, the supernatant was discarded, and fresh low calcium Tyrode's solution was added to wash the SAN. The SAN tissue was washed twice with low calcium Tyrode's solution and three times with Kraft-Bruhe medium containing (in mM): 100 potassium glutamate, 5 HEPES, 20 glucose, 25 KCl, 10 potassium aspartate, 2 MgSO_4_, 10 KH_2_PO_4_, 20 taurine, 5 creatine, 0.5 EGTA, and 1 mg/mL BSA (pH 7.4). The SAN tissue was maintained at 4°C for 2 hours, incubated at 37°C in the water bath for 10 min and then SAN cells were gently dissociated with sterile glass pipette. Dissociated SAN cells were used for experiments at room temperature (RT, 22-25°C) or 36 ± 0.5°C. For SAN culture isolations, all the tools were autoclaved and sterile for each use. All the solutions used were filtered using 0.22 μm filters.

#### Culture of adult mouse Sinoatrial node cells (SAN) and HEK cells

Glass coverslips (25 mm, size #0, Karl Hecht, Sondheim, Germany) were coated with 100x diluted laminin (Life Technologies, Grand Island, NY) and incubated for 4 hours at 37°C in 5% CO_2_. After 4 hours, coverslips were moved in a 24 well-plate (Falcon, Tewksbury, MA) and washed 3x with sterile PBS (in mM): 137 NaCl, 2.7 KCl, 10 Na_2_HPO_4_, 1.8 KH_2_PO_4_, pH = 7.4. Isolated SAN cells were resuspended in M1018 medium (10.7 g/L; Sigma-Aldrich, St Louis, MO) supplemented with 1x penicillin-streptomycin-glutamate (PSG), 4 mM NaHCO_3_, 10 mM HEPES, 10% fetal bovine serum (FBS). This media was used as is or was supplemented with either 10 mM 2,3-Butanedione 2-monoxime (BDM), 6.25 μM blebbistatin or 20 μM (S)-nitro blebbistatin. Resuspended cells were plated on laminin pre-coated glass coverslips and incubated for 4 hours at 37°C in 5% CO_2_, before the media was replaced with serum-free M1018 supplemented with the different contraction uncouplers (Reddy et al., 2018).

HEK cells were obtained from Sigma-Aldrich (cat #: ECACC 96121229). These cells are included in the European Collection of Authenticated Cell Cultures. HEK cells were cultured in DMEM supplemented with 1X pyruvate, 1X glutamax, 8% fetal bovine serum (FBS) and 5 mM glucose (without phenol red) at 37°C in a 5% CO_2_ incubator.

### Methods details

#### Infection of FRET biosensors and confocal imaging

For infection of adult mouse SAN and HEK cells, the serum-free M1018 media containing the different contraction uncouplers or DMEM media, respectively, were supplemented with 500 μL of the same media containing the different recombinant adenoviruses at 100 MOI each for 40 hours. Viruses were generated using the AdEasy system (Qbiogene, Inc.) (Luo et al., 2007). For imaging experiments, SAN cells were infected with Epac1-camp-based FRET biosensors (ICU3, also called cytosolic ICU3) or targeted sensors to the PM (PM-ICU3 – Lyn), SR (SR-ICU3 – PLB), myofilaments (MF-ICU3 – TnT) and nucleus (nuclear ICU3 – NLS) (Barbagallo et al., 2016; DiPilato and Zhang, 2009). Cells were incubated with the viruses at 37°C in 5% CO_2_ for 40 hours. Transduction efficiency ranged between 30-50%.

Immunofluorescence labeling was performed using freshly isolated SAN cells that were allowed to adhere to coverslips for 10 mins, and cultured SAN cells. Cells were fixed with 4% paraformaldehyde (PFA). Cells were then washed with phosphate-buffered saline (PBS; 3 x 10 mins). Cells were permeabilized for 20 mins with 0.1% Triton X-100% and then blocked with 5% donkey serum for 1 hour at RT. For experiments in Figure 1, the following primary antibodies were used to incubate cells overnight at 4°C: (1) monoclonal rat anti-HCN4 (1:300 dilution; Abcam ab32675) and (2) polyclonal goat anti-AC_VI_ (1:100 dilution; Santa Cruz sc68138). Cells were washed with PBS (3 x 10 mins) and then incubated with Alexa Fluor 555-conjugated donkey anti-rat (1:1000 dilution; Abcam ab150150) and Alexa Fluor 647-conjugated donkey anti-goat (1:1000 dilution; Abcam ab150135) secondary antibodies for 1 hour at RT. For experiments in Figure 4, cells infected with the different ICU3 biosensors were incubated with an anti-caveolin 3 antibody (1:200, Thermo-Fisher Scientific PA1-066) to label the PM, an anti-ryanodine receptor 2 (RyR2) antibody (1:200, Alomone Labs ARR-002) to label the SR, an Alexa Fluor 647 Phalloidin (1.3 U; Thermo-Fisher Scientific A22287) to label MF, or DAPI from the ProLong Diamond Antifade Mountant (Thermo-Fisher Scientific) to label the nucleus. Cells incubated with the anti-caveolin 3 and anti-RyR2 antibodies were further incubated with the secondary antibody Alexa Fluor 555-conjugated donkey anti-rabbit (1:1000 dilution; Thermo-Fisher Scientific A31572) following the same protocol as above. All the antibodies were diluted in blocking solution with 5% donkey serum. Cells were then washed with PBS (3 x 10 mins). Coverslips were mounted on the slides with ProLong Diamond Antifade Mountant (Thermo-Fisher Scientific) for subsequent visualization.

For immunofluorescent experiments in Figure 1, a Zeiss LSM700 laser scanning confocal microscope (Carl Zeiss, Oberkochen, Germany) equipped with a 405 nm, 488 nm, 555 nm and 647 nm lasers and paired with a Zeiss 63x oil immersion lens (numerical aperture = 1.4) was used to collect sequential images at different optical planes (z-axis steps: 0.4 μm) for HCN4 and AC6-associated fluorescent signal. For super resolution experiments in Figure 4, a Leica SP8 confocal microscope with high sensitivity detectors, a Plan Apo 63X 1.4 NA oil-immersion objective and a Lighting detector module was used. Z stacks were acquired for each sample. Processing of images with the Lighting detector module achieve a sub-diffraction limited resolution image of ∼120 nm. Images were acquired using a sequential protocol with appropriate wavelengths selected for the specific combination of fluorophores used in each sample. The upper and lower emission band thresholds were appropriately constrained to avoid/limit any bleed through. Identical settings and acquisition parameters were used for all specimens. Images were background subtracted, pseudo-colored, and analyzed offline. Line profile analysis was done using ImageJ (National Institutes of Health (NIH)), and Pearson's Correlation coefficient was perform using Imaris (Oxford Instruments) software.

#### FRET imaging and quantification

After infection and incubation of cells with FRET constructs for 40 hours, culturing media was replaced with serum and contraction uncoupler free media. The day of imaging, glass coverslips were transferred to glass bottom culture dishes (MatTek, Ashland, MA) containing 3 mL PBS at RT. A Leica DMI3000B inverted fluorescence microscope (Leica Biosystems, Buffalo Grove, IL) equipped with a Hamamatsu Orca-Flash 4.0 digital camera (Bridgewater, NJ) and controlled by Metafluor software (Molecular Devices, Sunnyvale, CA) acquired phase contrast, CFP480, and FRET ratio images. Phase contrast and CFP480 images were collected with 20x and 40x oil immersion objective lenses, while FRET ratio images were collected using only the 40x oil immersion objective lens. Images for FRET analysis were recorded by exciting the donor fluorophore at 430-455nm and measuring emission fluorescence with two filters (475DF40 for cyan and 535DF25 for yellow). All images were acquired every 20 s with exposure time of 300 ms for each channel and images were subjected to background subtraction. The donor/acceptor FRET ratio was calculated and normalized to the ratio value of baseline without stimulator. Averages of normalized curves and maximal response to stimulation were graphed based on FRET ratio changes. Experiments were performed at RT (22-25° C).

#### Electrophysiology

Spontaneous action potentials (APs) and AP firing frequencies in single freshly isolated and cultured adult mouse SAN cells were recorded using the perforated patch-clamp technique at 36 ± 0.5°C. Amphotericin B (240 μg/mL) was added into the pipette solution. Spontaneous APs were recorded in Tyrode's solution free of contraction uncouplers with the pipette filled with (in mM): 30 potassium aspartate, 10 NaCl, 10 HEPES, 0.04 CaCl_2_, 2.0 Mg-ATP, 7.0 phosphocreatine, 0.1 Na-GTP, pH 7.2. Data was acquired using an Axopatch 200B amplifier, Digidata 1440 digitizer and pClamp10 software (Molecular Devices). Recording electrodes were pulled from borosilicate capillary glass using a micropipette puller (Sutter Instruments model P-97, Novato, CA).

#### Cell size and shape measurements

Brightfield and epifluorescent images were acquired using a Leica DMI3000B inverted fluorescence microscope (Leica Biosystems, Buffalo Grove, IL) equipped with a Hamamatsu Orca-Flash 4.0 digital camera (Bridgewater, NJ) with 20x objectives. SAN cell size measurements were made using ImageJ software (National Institutes of Health (NIH), Bethesda, MD), with length measured from a polygon line through the center of each cell. Percentage of spindle-shaped cells was determined by calculating the number of elongated cells compared to all cells in each preparation. The objective is to differentiate between cell length and cell shape (e.g. spindle versus spherical) within a large population of freshly dissociated and cultured SAN cells obtained from the same field of view. This is because cell length may be similar between treatments, especially if only spindle-shaped cells are measured within the population of cells being analyzed. Conversely, the percentage of spindle-shaped cells may be reduced. Thus, providing these two independent measurements may provide further support to accept or reject the hypothesis that one treatment is more effective than another.

#### Transverse aortic constriction surgery

The survival surgeries were performed under general anesthesia using aseptic techniques. A small thoracotomy was done at the left sternal, second intercostal muscle. The thymus was separated, and the aorta visualized. A 27-gauge needle (0.4 mm in diameter) was placed transverse to the aorta and sterile suture was used to constrict the aorta to the size of the needle. Sham-operation was performed as described, but no ligation was performed. Muscles and skin were then sutured. A bolus injection of 0.1 mg/kg of buprenorphine SC was given for pain relief and every 12 hours after for 3 days. Mice were monitored daily until wound was healed. HF developed in Transverse Aortic Constriction (TAC) mice after 8 weeks.

#### Non-invasive echocardiographic imaging

Cardiac function in sham and HF mice were assessed using echocardiographic noninvasive imaging in a conscious state using VisualSonics Vevo 2100 (FUJIFILM VisualSonics, Inc., Toronto, ON, Canada). Mice were placed in a supine position on a heated echocardiography platform (37°C). The imaging generally lasted for approximately 5 minutes. The fractional shortening, cardiac dimensions, and intracardiac volume were obtained using M-Mode images. The measurements were performed in a blinded fashion, with papillary muscles used as a point of reference for consistency in the scan level. End diastole was defined as the maximal left ventricular (LV) diastolic dimension, and end-systole was defined as the peak of posterior wall motion. Fractional shortening (FS), a surrogate of systolic function, was calculated from LV dimensions as follows: FS = ((EDD-ESD)/EDD) x100%, where EDD and ESD are LV end-diastolic and end-systolic dimension, respectively.

#### Experimental design

The potential for variability in sample preparation, animals, ambiance conditions and other factors was accounted for by obtaining datasets from at least 2 different mice. All acquired data was included in the final analysis.

### Quantification and statistical analysis

#### Statistics and analysis

Data were analyzed using GraphPad Prism v6.0 (GraphPad Software, San Diego, CA), and expressed as mean ± SEM. The number of replicates is included in figure legends. GraphPad Prism Outlier Test was used to assess any potential outliers in the data. GraphPad was also used to assess normality of distribution. Statistical significance was determined using appropriate paired or unpaired two-tailed Student’s *t*-test, parametric or nonparametric tests and One-way analysis of variance (ANOVA) for multiple comparisons with appropriate post hoc test. Additional statistical details of experiments can be found in the figures and supplemental table legends, including the statistical test used and the exact P values. P < 0.05 was considered statistically significant (denoted by ∗ in figures).

## Data Availability

• All source data to generate all the figures are included in the Data S1 file. • This paper does not report original code. • Any additional information required to reanalyze the data reported in this paper is available from the lead contact upon request.

## Supplemental information

Document S1. Tables S1–S5

Data S1. Source data, related to Figures 1–6
