# Supplementary material for: Deciphering cellular signals in adult mouse sinoatrial node cells
Source: iScience. 2021 Dec 25;25(1):103693. doi: 10.1016/j.isci.2021.103693 (PMC8749457; doi:10.1016/j.isci.2021.103693)
Supplement: Document S1. Tables S1–S5 [file mmc1.pdf]

iScience, Volume *25*

Supplemental information

Deciphering cellular signals in

adult mouse sinoatrial node cells

Gopireddy R. Reddy, Lu Ren, Phung N. Thai, Jessica L. Caldwell, Manuela Zaccolo, Julie Bossuyt, Crystal M. Ripplinger, Yang K. Xiang, Madeline Nieves-Cintrón, Nipavan Chiamvimonvat, and Manuel F. Navedo

Table S1. SAN cell length and morphology related to Figure 1.

	length (µm)		% spindle-shaped cells	
**condition**	**0 hr**	**40 hr**	**0 hr**	**40 hr**
M1018	92.0 ± 3.0	27.6 ± 2.0*	80.6 ± 5.8	8.3 ± 1.2*
BDM	91.6 ± 2.9	35.5 ± 3.7*	78.9 ± 5.5	17.2 ± 2.2*
Bleb	86.1 ± 2.5	80.1 ± 3.2#	88.3 ± 4.1	75.6 ± 1.6#*
(S)-nitro bleb	97.0 ± 2.8	98.1± 2.9#	87.8 ± 1.4	89.4 ± 1.0#

Values are mean ± SEM. Significance was considered at $P < 0.05$. #Kruskal-Wallis test was used to compare cell length and % spindle-shaped cells within the 0 hours or 40 hours groups independently. For cell length dataset, $P > 0.9999$ for comparison between the 0 hours groups, except blebbistatin (Bleb) and (S)-nitro blebbistatin, which was 0.0514. $P > 0.9999$ and $P = 0.0806$ for comparison between 40 hours M1018 and BDM, and blebbistatin and (S)-nitro blebbistatin, respectively, and all other comparisons $P < 0.0001$. For % spindle-shaped cells dataset, $P > 0.9999$ for comparison between most 0 hours groups, except BDM and blebbistatin, which was 0.9201. P = 0.0003, 0.0249, 0.7199 and 0.0343 for comparison between 40 hours M1018 and (S)-nitro blebbistatin, BDM and (S)-nitro blebbistatin, blebbistatin and (S)-nitro blebbistatin, and M1018 and blebbistatin, respectively. *Mann-Whitney test was used to compare cell length and % spindle-shaped cells between the 0 and 40 hours datasets within the same group. For cell length dataset, $P < 0.0001$ for M1018 and BDM, 0.2033 for blebbistatin and 0.7766 for (S)-nitro blebbistatin. For % spindle-shaped cells dataset, $P = 0.0079$ for M1018, 0.0079 for BDM, 0.0397 for blebbistatin and 0.5317 for (S)-nitro blebbistatin.

Table S2. cAMP responses from $CAMPER_{CM}$ SAN cells related to Figure 2.

ligand	fresh cells ($\Delta R/R_0$)	culture cells ($\Delta R/R_0$)
forskolin	0.56 ± 0.03	0.53 ± 0.03
isoproterenol	-	0.23 ± 0.01*
histamine	-	0.24 ± 0.00*
CGRP	-	0.29 ± 0.01*
adenosine	-	0.23 ± 0.00*

Values are mean ± SEM. Significance was considered at $P < 0.05$. $^{\#}P = 0.295$ with Mann-Whitney test for comparison of forskolin responses in freshly dissociated and cultured $CAMPER_{CM}$ SAN cells. $^{*}P < 0.0001$ with One-way ANOVA with Bonferroni's post-test was used to compare all groups to forskolin responses.

Table S3. Compartmentalized cAMP responses in SAN cells related to Figure 4.

FRET biosensor	Pearson's Correlation	fsk + IBMX ($\Delta R/R_0$)	cAMP EC_{50} (nM)	iso ($\Delta R/R_0$)	fsk ($\Delta R/R_0$)	norm iso (% fsk)
cyt-ICU3	0.02 ± 0.04†	0.34 ± 0.01	71.4 ± 1.2	0.08 ± 0.00#	0.21 ± 0.01	39.8 ± 2.2
PM-ICU3	0.93 ± 0.01	0.37 ± 0.00	68.9 ± 1.2	0.09 ± 0.01#	0.19 ± 0.02	46.7 ± 4.2
SR-ICU3	0.89 ± 0.01	0.35 ± 0.01	65.1 ± 1.1	0.03 ± 0.00#	0.22 ± 0.00	15.3 ± 1.0*
MF-ICU3	0.90 ± 0.02	0.34 ± 0.01	77.3 ± 1.1	0.07 ± 0.00#	0.18 ± 0.00**	40.6 ± 1.1
nuclear-ICU3	0.92 ± 0.01	0.34 ± 0.00	72.5 ± 1.1	0.03 ± 0.00#	0.18 ± 0.00**	16.2 ± 0.8*

Values are mean ± SEM. Significance was considered at $P < 0.05$. †Kruskal-Wallis with Dunn's multiple comparisons test was used to examine statistical differences in Pearson's correlation coefficient between non-target and targeted sensors. $P \leq 0.0001$ between cytosol, PM and nucleus. $P = 0.0251$ between cytosol and SR and 0.0175 between cytosol and MF. ††Kruskal-Wallis with Dunn's multiple comparisons test was used to compare fsk + IBMX responses. $P > 0.9999$ for comparisons between cytosol/SR, cytosol/MF, cytosol/nucleus, PM/SR, SR/MF, SR/nucleus and MF/nucleus. $P = 0.0957$ between cytosol/PM, 0.2297 between PM/MF, and 0.0814 between PM/nucleus. †††extra sum-of-squares F test. $P = 0.9463$. #Mann-Whitney test was used for comparison between the isoproterenol (iso) and forskolin (fsk) responses for each biosensor. $P = 0.0016$ for cytosol, 0.0079 for PM, 0.0010 for SR, 0.0016 for MF and 0.0002 for nucleus. **Kruskal-Wallis with Dunn's multiple comparisons test was used to assess statistical differences in maximal fsk response between all biosensors. $P > 0.9999$ for comparisons between cytosol/PM, cytosol/SR, PM/MF, PM/nucleus and MF/nucleus. $P = 0.0974$ between cytosol and MF, 0.1042 between cytosol and nucleus, 0.6648 between PM and SR, 0.0034 between SR and MF, and 0.0020 between SR and nucleus. *One-way ANOVA with Tukey's multiple comparisons test was used to assess statistical differences in normalized iso responses between the different groups. $P < 0.0001$ for comparisons of cytosol, PM and MF groups with SR and nucleus groups. $P = 0.172$ between cytosol and PM, 0.9979 between cytosol and MF, 0.1905 between PM and MF, and 0.9973 between SR and nucleus.

Table S4. Signaling network responses in SAN cells related to Figure 5.

ligand	cGI500 ($\Delta R/R_0$)	AKAR3 ($\Delta R/R_0$)	Camui ($\Delta R/R_0$)	DKAR ($\Delta R/R_0$)
ANP	0.10 ± 0.01	-	-	-
SNAP	0.20 ± 0.01*	-	-	-
isoproterenol	-	0.15 ± 0.01	-	-
forskolin	-	0.25 ± 0.02*	-	-
phenylephrin e	-	-	0.10 ± 0.01	-
calcium	-	-	0.20 ± 0.01*	-
histamine	-	-	-	0.11 ± 0.01
PDBu	-	-	-	0.19 ± 0.01*

Values are mean ± SEM. Significance was considered at $P < 0.05$. *Mann-Whitney test was used to compared between responses for a given biosensor. $P = 0.0002$ for comparison of ANP and SNAP responses with the cGI500 sensor, 0.0123 for comparison of iso and fsk responses with the AKAR3 sensor, 0.0006 for comparison of PE and calcium responses with the Camui, and 0.0022 for comparison of hist and PDBu responses with the DKAR sensor.

Table S5. Cell length and morphology, and cAMP and cGMP signaling in sham and HF SAN cells related to Figure 6.

	length (µm)		% spindle-shaped cells		FRET biosensors ($\Delta R/R_0$)	
**condition**	**0 hr**	**40 hr**	**0 hr**	**40 hr**	**CUTie (%)**	**cGI500 (%)**
sham	100.1 ± 2.4	99.5 ± 3.3	86.1 ± 4.2	83.3 ± 3.3	48.2 ± 0.9	34.9 ± 1.5
HF	87.4 ± 1.8*	87.1 ± 2.4*	82.8 ± 1.8	77.8 ± 4.4	26.0 ± 1.7#	22.0 ± 1.9#
wild type (M1018)	-	27.6 ± 2.0#	-	8.3 ± 1.2#	-	-

Values are mean ± SEM. Significance was considered at $P < 0.05$. *Kruskal-Wallis test for comparison between all groups. For comparison of cell length datasets, $P > 0.9999$ between sham and HF cells at 0 and 40 hours. $P = 0.0018$, 0.0016, 0.0203, 0.016 for comparison of sham and HF at 0 hours, sham 0 hours and HF 40 hours, sham 40 hours and HF 0 hours, and sham and HF at 40 hours. For % spindle-shaped cells, $P > 0.9999$ for all comparisons. #Mann-Whitney was used to compare cell length ($P < 0.0001$) and % spindle-shaped cells ($P < 0.0079$) between the wild type cells in M1018 and HF cells in (S)-nitro blebbistatin, as well as to compare results from the FRET biosensor experiments in sham and HF cells ($P < 0.0001$).
